# Cardiorespiratory kinetics in exercise physiology: estimates and predictions using randomized changes in work rate

**DOI:** 10.1007/s00421-021-04878-z

**Published:** 2021-12-28

**Authors:** Uwe Hoffmann, Felix Faber, Uwe Drescher, Jessica Koschate

**Affiliations:** 1 Department of Exercise Physiology, Institute of Exercise Training and Sport Informatics, German Sport University Cologne, Am Sportpark Müngersdorf 6, 50933 Cologne, Germany; 2 Geriatric Medicine, Department for Health Services Research, School of Medicine and Health Sciences, Carl Von Ossietzky University Oldenburg, Ammerlaender Heerstr.140, 26129 Oldenburg, Germany

**Keywords:** Exercise testing, PRBS, Kinetics, Prediction

## Abstract

**Purpose:**

Kinetics of cardiorespiratory parameters (CRP) in response to work rate (WR) changes are evaluated by pseudo-random binary sequences (PRBS testing). In this study, two algorithms were applied to convert responses from PRBS testing into appropriate impulse responses to predict steady states values and responses to incremental increases in exercise intensity.

**Methods:**

13 individuals (age: 41 ± 9 years, BMI: 23.8 ± 3.7 kg m^−2^), completing an exercise test protocol, comprising a section of randomized changes of 30 W and 80 W (PRBS), two phases of constant WR at 30 W and 80 W and incremental WR until subjective fatigue, were included in the analysis. Ventilation ($\dot{V}_{{\text{E}}}$), O_2_ uptake ($\dot{V}{\text{O}}_{2}$), CO_2_ output ($\dot{V}{\text{CO}}_{2}$) and heart rate (HR) were monitored. Impulse responses were calculated in the time domain and in the frequency domain from the cross-correlations of WR and the respective CRP.

**Results:**

The algorithm in the time domain allows better prediction for $\dot{V}{\text{O}}_{2}$ and $\dot{V}{\text{CO}}_{2}$, whereas for $\dot{V}_{{\text{E}}}$ and HR the results were similar for both algorithms. Best predictions were found for $\dot{V}{\text{O}}_{2}$ and HR with higher (3–4%) 30 W steady states and lower (1–4%) values for 80 W. Tendencies were found in the residuals between predicted and measured data.

**Conclusion:**

The CRP kinetics, resulting from PRBS testing, are qualified to assess steady states within the applied WR range. Below the ventilatory threshold, $\dot{V}{\text{O}}_{2}$ and HR responses to incrementally increasing exercise intensities can be sufficiently predicted.

## Introduction

The response of cardio-respiratory parameters (CRP), such as oxygen uptake ($\dot{V}{\text{O}}_{2}$), ventilation ($\dot{V}_{{\text{E}}}$), CO_2_ output ($\dot{V}{\text{CO}}_{2}$) and heart rate (HR) to changes in work rate (WR) are measured during cardio-pulmonary exercise tests. From these data, the regulation of aerobic metabolism, ventilation and the cardio-vascular system can be derived (see Poole and Jones 2012 for a comprehensive overview). In terms of control technology, the CRP responses to exercise can be described as a single-input/single-output system (i. e. WR-CRP). The description of these systems allows to identify the influence of factors such as type of exercise (Koschate et al. 2019a, b), ambient conditions (Drescher et al. 2018) and individual characteristics, e. g. age (Koschate et al. 2016a, b; Ebine et al. 2018; Patti et al. 2021) or fitness (Beltrame et al. 2020; Inglis et al. 2021), on the regulation of the cardiorespiratory system. From the individual kinetics and other characteristics of regulation, interventions, e. g. exercise prescriptions for athletes and patients, can be defined. Furthermore, the combination of exercise tests with information from other non-invasive methods to assess cardiovascular data, e. g. continuous blood pressure measurements, cardiac output and pulse-oximetry, can improve the diagnostic outcome (Oyake et al. 2021).

Typically, kinetics are analyzed and described using step responses in combination with data fitting procedures to approximate single mono-exponential functions or sums thereof (Whipp and Rossiter 2005; Keir et al. 2014; Murias et al. 2011). The disadvantage of these methods is twofold: (1) Due to an unfavourable signal-to-noise ratio, several repetitions of the WR steps are required, which extends the test duration significantly and may necessitate more than one test session. This may exclude specific groups of subjects, as older persons, persons with limited endurance, or persons with a tight schedule, from testing. (2) This kind of modelling requires the assumption of an explicit model with fixed parameters, e. g. a first-order system with time delay (e.g. Ma et al. 2010). The model parameters are determined by iterative least-square criteria and steady states are mandatory. However, if responses to increases (on-step) and decreases in WR (off-steps) are evaluated separately (e.g. Özyener et al. 2001; Fukuoka et al. 2002), potential asymmetries indicate objections to the assumption of dynamic linearity to describe the system response. Changes in step amplitudes may result in differences for the model parameters which is in contradiction to dynamic linearity.

Another approach excites the CRP by WR changes via pseudo-random binary sequences (PRBS) (Hoffmann et al. 2013). This approach requires dynamic linearity between WR and the analyzed CRP, but no explicit model. One PRBS sequence consists of *Z* intervals that remain constant for a fixed time $\delta$ and are pseudo randomly assigned to changes between two input levels, i.e. WR levels. The resulting sequence with a duration *δ Z* will be repeated and analysed by time-series analyses in terms of auto- and cross-correlation functions (ACF, CCF). The periodic ACF of the PRBS approximates a periodic impulse function with the periodicity *δ Z*. In the frequency domain the power spectrum remains flat over a chosen range of frequencies (see Khoo 2018, p. 250ff for a detailed overview).

*Z* and $\delta$ can be adjusted to concentrate the exciting frequency range to the corresponding frequencies of the CRP dynamics (Seborg et al. 2016, p. 113) and, therefore, the test can be adapted to the given process. For the description of a linear, time-invariant (LTI-) single-input/single-output system the resulting CCF of the input, i.e. WR, and the output, i.e. a CRP, allows a kinetics description in both the time and the frequency domain. The resulting non-parametric description is strongly recommended, when the “…actual model order or time delay is unknown…” and the dynamic behaviour “…cannot be described by standard low-order models” (Seborg et al. 2016, p. 373f).

Both analyses can be summarized by an impulse response function *h*(*t*). This function *h*(*t*), in combination with any input signal *x*(*t*), allows a prediction of the output *y*(*t*) by applying the convolution integral for any LTI-system:

1 $$y\left(t\right)={\int }_{-\infty }^{\infty }h\left(\lambda \right) x\left(t-\lambda \right) d\lambda$$

While *h*(*t*) can easily be derived for parametric models, e. g. single mono-exponential functions, the conversion of the resulting CCF from a PRBS test is more complex.

In the following, the application of two different algorithms to convert the CCF of WR-CRP from a PRBS testing into an appropriate impulse response *h*(*t*) will be demonstrated. We hypothesize that steady states and the response to incremental WR can be predicted by convolution with the impulse response.

This attempt also reduces the complications caused by the signal-to-noise ratio for the CRP and, by that, reduces the testing time significantly.

## Methods

### Subjects, test protocol and instrumentation

The data for this analysis were taken from the baseline tests of the four missions of campaign four inside the Human Exploration Research Analogue (HERA) facility at NASA Johnson Space Center (see Koschate et al. 2021 for details). Ethical approval was obtained from the Institutional Review Board at the NASA Johnson Space Center (Protocol number: Pro2320) as well as the Ethical Committee of the German Sport University Cologne (Protocol number: 074/2016). Written informed consent was derived from all participants prior to the experiments.

**Fig. 1:**
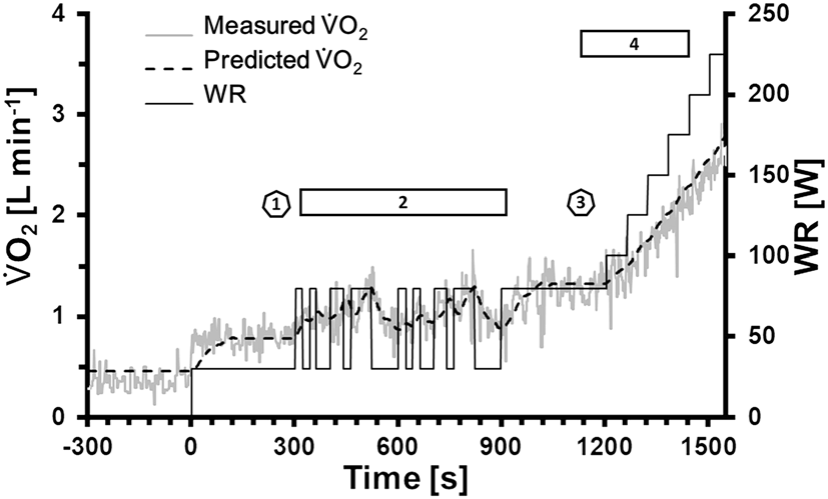
WR protocol, individual measured $\dot{V}{\text{O}}_{2}$ and predicted $\dot{V}{\text{O}}_{2}$ response from the calculated impulse response *h*_TD_(*t*) (see text). 1: interval to calculate the mean at 30 W; 2: PRBS interval for *h*_TD_(*t*) calculation; 3: interval to calculate the mean at 80 W; 4: interval to calculate the response to incrementally increansing WR. *WR* work rate; $\dot{V}O_{2}$ oxygen uptake. Differences for predictions from *h*_TD_(*t*) and *h*_FD_(*t*) were not visible for this subject

13 subjects (age: 41 ± 9 years, BMI: 23.8 ± 3.7 kg m^−2^) were included in the analysis. The tests were performed on a cycle ergometer in the upright body position, using a complex exercise protocol, which comprised a section with randomized changes between 30 and 80 W (PRBS), two phases of constant WR at 30 W and 80 W and finally an incremental test until subjective fatigue (see Fig. 1). The PRBS consisted of two identical sequences of 300 s. Each sequence was divided into $Z=15$ intervals of $\delta =20 \mathrm{s}$ which were pseudo-randomly assigned to 30 W or 80 W.

During the exercise test, $\dot{V}{\text{O}}_{2}$, $\dot{V}{\text{CO}}_{2}$ and $\dot{V}_{{\text{E}}}$ were measured breath by breath using a metabolic cart (Metalyzer 3B, Cortex Biophysik GmbH, Leipzig, Germany). In addition, beat-to-beat HR was obtained using a wireless ECG belt system (CustoGuard 3, Customed, Ottobrunn, Germany). A constant sampling period $t=1s$ was realized by synchronizing and interpolating all input (WR) and CRP output data ($\dot{V}{\text{O}}_{2}$, $\dot{V}{\text{CO}}_{2}$, $\dot{V}_{{\text{E}}}$, HR). Breath-by-breath data were interpolated stepwise and beat-to-beat data linearly.

### Calculation of kinetics response from data in the PRBS interval

**Fig. 2:**
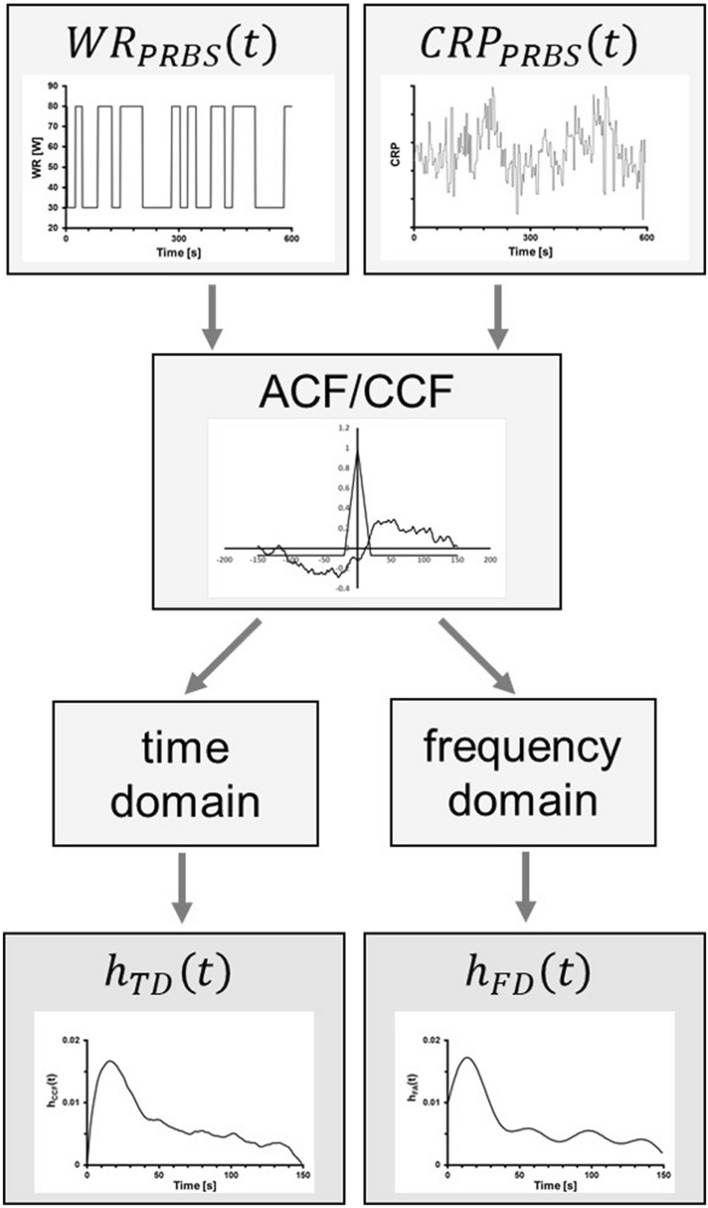
Obtaining an impulse response function from both, time domain (*h*_TD_(*t*)) and frequency domain (*h*_FD_(*t*)) analysis, respectively. Starting with PRBS changes in WR and the related CRP response, the CCF is calculated and converted into *h*_TD_(*t*)) and *h*_FD_(*t*)

The ACF ${\Phi }_{xx}(t)$ and CCF ${\Phi }_{xy}(t)$ were derived, using the PRBS samples of the WR as input signal *x*(*t*), i. e. WR_PRBS_(*t*), and the corresponding CRP data as output *y*(*t*), i. e. CRP_PRBS_(*t*). As illustrated in Fig. 2, two different approaches were taken to get impulse response functions,${\tilde{h }}_{\mathrm{TD}}\left(t\right)$ and ${\tilde{h }}_{\mathrm{FD}}\left(t\right)$, respectively:

First, in the time domain (TD) the impulse response function ${\tilde{h }}_{\mathrm{TD}}\left(t\right)$ was calculated according to Bo et al. (2006):

2 $${\tilde{h }}_{\mathrm{TD}}\left(t\right)= \left\{\begin{array}{c} \frac{{\Phi }_{xy}\left(t\right)-{\Phi }_{xy}\left(-t\right)}{C} \quad \textrm{for} \quad\delta \le t \\ \\ \frac{{\Phi }_{xy}\left(t\right)-{\Phi }_{xy}\left(-t\right)}{\left(\frac{1}{2}+\frac{t}{\delta } \left(1-\frac{t}{2\delta }\right)\right)C}\quad \textrm{for} \quad 0<t<\delta \\ \\ \frac{{2 [\Phi }_{xy}\left(0\right)-{\Phi }_{xy}(-\delta )] }{C}\quad \textrm{for} \quad t=0 \\ \end{array}\right.$$

where $$C=\left(1+\frac{1}{Z}\right)\delta$$ and $$0\le \mathrm{ t }<\frac{\updelta Z}{2}$$.

For the second approach, the correlation functions were converted into frequency domain signals, using Fast Fourier transformation. Since the signal power mainly distributes on a few low frequencies for the sequence chosen, the cut-off frequency was determined to be half the PRBS shift frequency (Uhrig 1970, p. 297). Thus, a lowpass filter with passband frequency 25 mHz, which corresponds to the seventh harmonic frequency of this sequence, was applied to the spectral-density functions ${\Phi }_{xx}(j\omega )$ and ${\Phi }_{xy}(j\omega )$. Next, the transfer function ${H}_{\mathrm{FD}}(j\omega )$ was calculated according to the known input–output relation in the frequency domain:

3 $${H}_{\mathrm{FD}}\left(j\omega \right)=\frac{{\Phi }_{xy}\left(j\omega \right)}{{\Phi }_{xx}\left(j\omega \right)}$$

Applying the inverse Fast Fourier transformation to ${H}_{\mathrm{FD}}(j\omega )$, a second impulse response function ${\tilde{h }}_{\mathrm{FD}}\left(t\right)$ was obtained.

For ${\tilde{h }}_{\mathrm{TD}}\left(t\right)$ the valid range is limited to $0\mathrm{ s}\le t <150 s$ due to the composition of the PRBS. Although, ${\tilde{h }}_{\mathrm{FD}}\left(t\right)$ is defined for a range $0\le t <300 s$, for the following calculations only the range $0\mathrm{ s}\le t <150 s$ was considered, assuming that under physiological conditions the response is completed after 150 s. To get a comparable basis, $\tilde{h }\left(t\right)$ was normalized through its sum over the range $0\mathrm{ s}\le t <150 s$:

4 $$h\left(t\right)= \frac{\tilde{h }\left(t\right)}{{\sum }_{0}^{149}\tilde{h }\left(t\right)} \quad \textrm{for} \quad 0\le t<150$$

In the last step, the normalized impulse response functions ${h}_{\mathrm{TD}}\left(t\right)$ and ${h}_{\mathrm{FD}}\left(t\right)$, must be converted into a relation of WR and the CRP of interest. By convolution of the input ${\mathrm{WR}}_{\mathrm{PRBS}}\left(t\right)$ with the normalized response function *h*(*t*)

5 $${\widehat{\mathrm{WR}}}_{\mathrm{PRBS}}\left(t\right)={\int }_{-\infty }^{\infty }h\left(\lambda \right) {\mathrm{WR}}_{\mathrm{PRBS}}\left(t-\lambda \right) d\lambda$$

and *a* following linear regression with ${\widehat{\mathrm{WR}}}_{\mathrm{PRBS}}$ and ${\mathrm{CRP}}_{\mathrm{PRBS}}$ the coefficients gain *a* and offset *b* are obtained. Therefore, the predictions ${\mathrm{Pred}}_{\mathrm{TD}}\left(t\right)$ and ${\mathrm{Pred}}_{\mathrm{FD}}\left(t\right)$ as a response to any WR are given for each CRP of interest by:

6 $${\mathrm{Pred}}_{x}\left(t\right)=a{\int }_{-\infty }^{\infty }h\left(\lambda \right) \mathrm{WR}\left(t-\lambda \right) d\lambda +b$$

where $h\left(t\right)$ represents ${h}_{\mathrm{TD}}\left(t\right)$ and ${h}_{\mathrm{FD}}\left(t\right)$, respectively, and the regression parameters *a* and *b* vary with TD, FD, and each CRP.

### Data analysis

The measured data ($\dot{V}{\text{O}}_{2}$, $\dot{V}{\text{CO}}_{2}$, $\dot{V}_{{\text{E}}}$, HR) as well as the predictions ${\mathrm{Pred}}_{\mathrm{TD}}\left(t\right)$ and ${\mathrm{Pred}}_{\mathrm{FD}}\left(t\right)$ were individually averaged for the last 30 s of the constant WR phases at 30 W and 80 W (refer to Fig. 1). For the PRBS interval and the phase of incremental WR from 80 to 175 W, the Pearson correlation coefficients of measured and predicted data were individually calculated (*r*_PRBS_, *r*_80–175 W_).

Relative residuals Res(*t*) were individually calculated by:

7 $$\mathrm{Res}\left(t\right)=\frac{{\mathrm{Pred}}_{\mathrm{TD}}\left(t\right)-\mathrm{CRP}\left(t\right)}{\mathrm{CRP}\left(t\right)}$$

for each second and each CRP. For the PRBS interval and the phase of incremental exercise (80–175 W) individual means (Res_ME_), the related standard error (Res_SE_), and the individual correlation coefficients (Res_*r*_) between Res(*x*) and measured data were calculated.

### Statistical analyses

For the comparisons of *r*_PRBS_ and *r*_80–75 W_ resulting from Pred_TD_ and Pred_FD_ paired *t*-tests were applied. Two-way ANOVA for repeated measurements was used to analyse the steady states (factors: WR and method) as well as Res_ME_, Res_SE_ and Res_*r*_ with the factors “interval” (PRBS interval and the phase of incremental exercise (80–175 W)) and “parameter” ($\dot{V}{\text{O}}_{2}$, $\dot{V}{\text{CO}}_{2}$, $\dot{V}_{{\text{E}}}$, HR). In case of significant effects, post-hoc Bonferroni-tests were applied to compare single means. Level of significance was set to *P* ≤ 0.05.

## Results

Figure 1 shows an individual example for a calculation of $\dot{V}{\text{O}}_{2}$ in response to the complete exercise protocol.

**Fig. 3:**
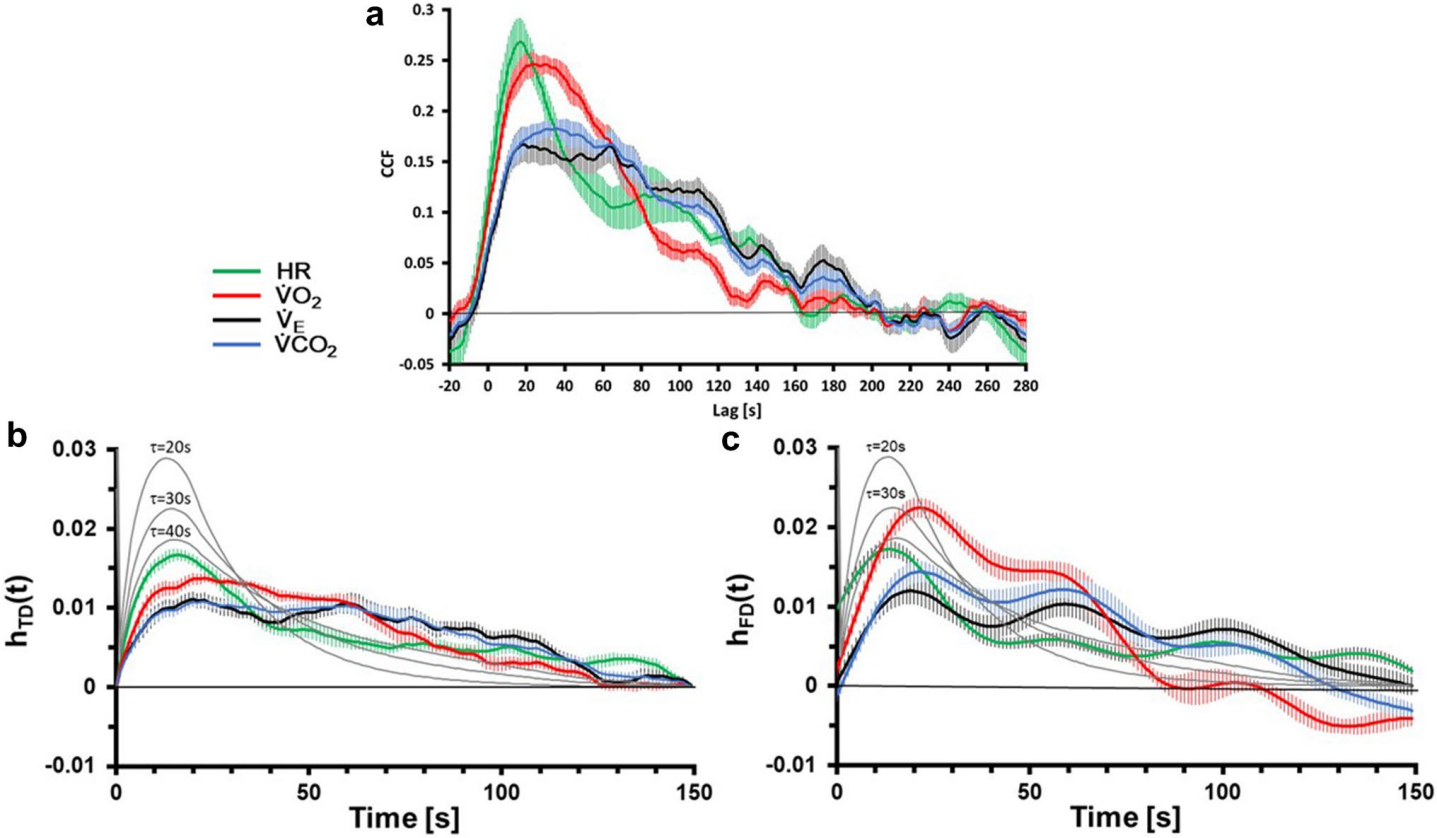
Cross-correlation function (CCF) (**a**) and the resulting impulse responses *h*_TD_(*t*) (**b**), *h*_FD_(*t*) (see text for details) (**c**) for the different cardiorespiratory parameters (means ± SE, *N* = 13). The grey lines in the background of b/c indicate theoretical responses of first-order systems with time constants *τ* = 20 s, *τ* = 30 s and *τ* = 40 s calculated with these algorithms

The CCF data from the PRBS phase and the resulting impulse responses *h*_TD_(*t*), *h*_FD_(*t*) are given in Fig. 3. The time courses for the different CRP differ significantly between the two algorithms (Fig. 3b, c). In contrast to a theoretical first-order model with an instantaneous increase to the maximum and an exponential decrease, the smooth increase (indicated as grey line in Fig. 3b, c), visible for the first seconds (0 < *t* ≤ 10 s), had to be expected, considering the characteristics of the CCF. The averaging over the individual results with potential time-delays may result in a further smoothing of the signal. In this interval (0 < *t* ≤ 10 s), *h*_TD_(*t*) of HR showed the fastest response followed by the responses of $\dot{V}{\text{O}}_{2}$ and, similarly, $\dot{V}_{{\text{E}}}$ and $\dot{V}{\text{CO}}_{2}$. For *h*_FD_(*t*), different observations can be summarized: *h*_FD_(*t*) of HR started less steep and the maximum for *h*_FD_(*t*) of $\dot{V}{\text{O}}_{2}$ is much higher compared to *h*_TD_(*t*) of $\dot{V}{\text{O}}_{2}$.

All impulse responses (Fig. 3b, c) calculated from the CCF (Fig. 3a) showed a slow decrease towards *t* = 150 s. Only for HR a steep decrease can be observed after the maximum until approximately *t* = 40 s. While *h*_TD_(*t*) is zero by definition at *t* = 0 and *t* = 150 s, whereas *h*_FD_(*t*) shows deviations from 0.

The predicted steady-state calculations for 30 W and 80 W were compared with averages from measured data (Table 1). Both algorithms predicted higher 30 W and lower 80 W steady states for all parameters. Remarkably, the highest absolute differences were found for $\dot{V}{\text{O}}_{2}$ predicted via Pred_FD_. Applying the impulse in the time domain (Pred_TD_), $\dot{V}{\text{O}}_{2}$ levels at 30 W and 80 W can be predicted with small deviations from measured data (− 3%, 1%).

**Table 1:** Characteristics for the estimated data and the results from steady-state analysis

Parameter	Method	30 W		80 W		*r*_PRBS_	*r*_80–175 W_
		mean ± SD	∆	mean ± SD	∆		
HR [min^−1^]	SteadyState	91 ± 11		120 ± 16			
	Pred_TD_	95 ± 13	− 4%	115 ± 15	4%	0.64 ± 0.12	0.91 ± 0.09
	Pred_FD_	94 ± 13	− 3%	116 ± 15	4%	0.68 ± 0.11	0.91 ± 0.09
	Significance	a, c		a, b, c		c	
$\dot{V}_{{\text{E}}}$ [L min^−1^]	SteadyState	21.0 ± 2.5		32.6 ± 2.5			
	Pred_TD_	22.2 ± 2.2	− 6%	31.2 ± 2.6	4%	0.45 ± 0.10	0.78 ± 0.14
	Pred_FD_	22.4 ± 1.9	− 7%	31.0 ± 2.7	5%	0.47 ± 0.08	0.78 ± 0.14
	Significance	b		a, b			
$\dot{V}{\text{O}}_{2}$ [L min^−1^]	SteadyState	0.71 ± 0.07		1.18 ± 0.08			
	Pred_TD_	0.73 ± 0.07	− 3%	1.17 ± 0.09	1%	0.62 ± 0.09	0.82 ± 0.07
	Pred_FD_	0.80 ± 0.07	− 12%	1.08 ± 0.09	8%	0.59 ± 0.09	0.82 ± 0.07
	Significance	b, c		b, c		c	c
$\dot{V}{\text{CO}}_{2}$ [L min^−1^]	SteadyState	0.62 ± 0.08		1.09 ± 0.08			
	Pred_TD_	0.66 ± 0.09	− 7%	1.05 ± 0.1	4%	0.57 ± 0.09	0.86 ± 0.06
	Pred_FD_	0.7 ± 0.07	− 13%	1.01 ± 0.08	8%	0.55 ± 0.09	0.86 ± 0.06
	Significance	b, c		b, c		c	c

SteadyState: individual average over 30 s for measured CRP; Pred_TD_: Prediction calculated from *h*_TD_(*t*); Pred_FD_: Prediction calculated from *h*_FD_(*t*); *r*_PRBS_: individual correlation coefficient for measured and predicted data of the PRBS interval; *r*_80–175 W_: individual correlation coefficient for measured and predicted data of the incremental exercise interval

a: SteadyState vs. Pred_TD_

b: SteadyState vs. Pred_FD_

c: Pred_TD_ vs. Pred_FD_

Another criterion to evaluate the algorithm can be derived from the correlations between measured and predicted values (*r*_PRBS_, *r*_80–175 W_). Significant differences were found for the two prediction algorithms (*P* < 0.05) for HR (*r*_PRBS_ only), $\dot{V}{\text{O}}_{2}$ and $\dot{V}{\text{CO}}_{2}$ (Table 1). The highest correlations for *r*_PRBS_ were found for HR followed by $\dot{V}{\text{O}}_{2}$. $\dot{V}{\text{CO}}_{2}$ showed slightly lower values compared to $\dot{V}{\text{O}}_{2}$ while $\dot{V}_{{\text{E}}}$ showed the lowest correlations. The wider data range of the CRP data for the increasing WRs compared to the PRBS is the explanation that coefficients *r*_80–175 W_ were higher than *r*_PRBS_ in all cases. It can be noted that the ranking between $\dot{V}{\text{O}}_{2}$ and $\dot{V}{\text{CO}}_{2}$ changed for *r*_80–175 W_.

**Fig. 4:**
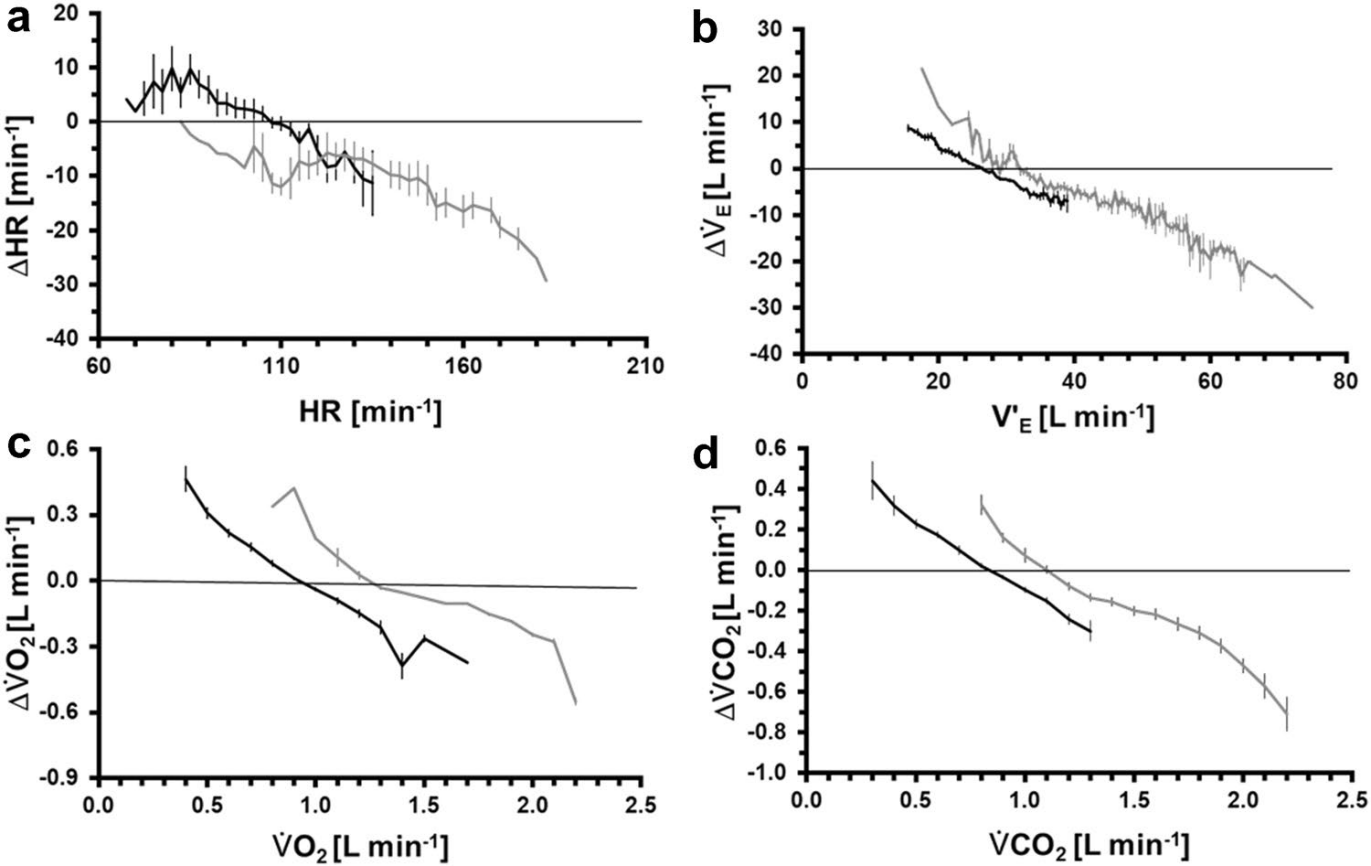
Differences ∆ (= Pred_TD_—measured data) for the different CRP related to the rounded measured values for the PRBS phase (black lines) and in the range for 80–175 W (grey lines) (means ± SE, *N* = 13)

A more detailed analysis can be derived from the residuals as differences between predicted and measured data (Table 2 and Fig. 4 for Pred_TD_). The relative residuals (Res_ME_) and standard errors of these (Res_SE_) (see Eq. 7) differ between HR and $\dot{V}_{{\text{E}}}$, $\dot{V}{\text{O}}_{2}$, as well as $\dot{V}{\text{CO}}_{2}$. For all CRP, the measured data from increasing WR (80–175 W) were significantly underestimated according to the individual means. However, in most cases remarkable correlations between the residual (measured—Pred_TD_) were found. The course of residuals (Fig. 4) indicates another aspect for the quality of the predictions: In most cases, systematic decreases of ∆ with increasing values of the respective parameter are visible. Only for HR, a range of stagnation can be identified for lower HR and in the 80–175 W interval.

**Table 2:** Analysis of relative residuals (Res) between Pred_TD_ and measured data

			HR	$\dot{V}_{\text{E}}$	$\dot{V}{\text{O}}_{2}$	$\dot{V}{\text{CO}}_{2}$
Res_ME_	PRBS	$\bar{x}$	− 0.002	− 0.008	− 0.009	− 0.009
		*s*	0.001	0.005	0.005	0.005
			a			
	80–175 W	$\bar{x}$	− 0.071	− 0.115	− 0.037	− 0.103
		*s*	0.049	0.062	0.041	0.055
					a	
Res_SE_	PRBS	$\bar{x}$	− 0.002	− 0.005	− 0.005	− 0.005
		*s*	0.001	0.001	0.001	0.001
			a			
	80–175 W	$\bar{x}$	− 0.002	− 0.007	− 0.006	− 0.006
		*s*	0.001	0.002	0.001	0.002
			a			
Res_*r*_	PRBS	$\bar{x}$	0.753	− 0.762	− 0.887	− 0.794
		*s*	0.100	0.077	0.050	0.072
				a	b	
	80–175 W	$\bar{x}$	− 0.248	− 0.533	− 0.827	− 0.790
		*s*	0.472	0.219	0.137	0.145
			c		d	

Mean (Res_ME_), standard error (Res_SE_) and correlation (Res_*r*_) were calculated individually for each CRP in intervals PRBS and 80–175 W. The results were analysed by two-way ANOVA and post-hoc comparisons

^a^*P* < 0.05 for all other CRP

^b^$$\dot{V}{\text{O}}_{2}$$-$$\dot{V}{\text{CO}}_{2}$$: *p* < 0.05

^c^HR-$$\dot{V}{\text{CO}}_{2}$$, HR-$$\dot{V}_{{\text{E}}}$$: *p* < 0.05

^d^$$\dot{V}_{{\text{E}}}$$-$$\dot{V}{\text{O}}_{2}$$, $$\dot{V}{\text{O}}_{2}$$-$$\dot{V}{\text{CO}}_{2}$$: *p* < 0.05

$$ \bar{x} $$, mean

*s*, standard deviation

## Discussion

The results of the applied analyses demonstrate the possibility to describe the kinetics of the CRP by an impulse response, and the predictability of CRP steady states as well as responses to increasing WR within certain limits from the PRBS data assessed applying moderate WR intensities. The quality of analysis and prediction must be evaluated separately for each CRP. For all parameters of interest, contradictions to dynamic linear models were identified.

### The algorithms

Both analytical approaches (*h*_TD_, *h*_FD_) assume dynamic linearity between WR and the respective CRP, since they were calculated from the CCF for the respective CRP. Hence, the mentioned contradictions to the assumptions of dynamic linearity might introduce uncertainties regarding the reliability of the predictions. However, the applied analytical approaches allow to avoid the assumption of a specific model, as used e. g. for exponential fits. However, as demonstrated in Fig. 3, the resulting impulse response may be compared to explicit, e.g. first-order models.

The application of the CCF in conjunction with randomized WR changes of 30 W and 80 W has a disadvantage: The PRBS chosen (15 intervals, 20 s each, total duration 300 s per sequence) has a limited frequency bandwidth. Therefore, rapid changes cannot be analysed. Consequently, in the frequency domain, the analysis must be restricted to the first seven harmonics (period length of 43 s) of the Fast Fourier transformation (Eq. 3) and its inverse (Uhrig 1970, p. 297). This explains the transient increase in the first few seconds of the impulse response for both, *h*_TD_ and *h*_FD_. Even in a theoretical first-order system a mono-exponential decrease, as demonstrated in Fig. 3, cannot be identified. The analysis of simulated responses of first-order systems do not show the typical rapid increase at *t* = 0 s and the exponential decrease (see also Fig. 3).

$\dot{V}{\text{O}}_{2}$ showed significant differences between *h*_TD_(*t*) and *h*_FD_(*t*). This difference is the result of the applied normalisation (Eq. 4). While the *h*_TD_ (*t*) returns to zero at *t* = 150 s, this must not be true for *h*_FD_(*t*). For *h*_FD_(*t*) of $\dot{V}{\text{O}}_{2}$ there was a significant undershoot (see Fig. 3, *t* > 110 s). This results in a small normalization coefficient ${\sum }_{0}^{149}{\tilde{h }}_{i}\left(t\right)$ and an amplified *h*_FD_(*t*). As stated in the methods section, for *h*_FD_(*t*) the impulse response was cut at *t* > 150 s. The reason for this cut is that for *t* > 150 s no further physiological origin can be expected as impulse response. However, for some single data sets, calculations were performed with *h*_FD_(*t*) in the range 0 < *t* < 300 s but these analyses yielded non-reasonable and inconsistent results, with higher Res_ME_. Hence, the applied algorithm in the frequency domain must be regarded as less qualified for the prediction (Pred_FD_(*t*)). The further discussion will be focussed on the Pred_TD_ (*t*) for $\dot{V}{\text{O}}_{2}$ and the other CRP.

### Predictability for the CRP responses

The predictability for the different CRP during moderate exercise, i. e. between 30 and 80 W, can be ranked by means of different criteria and aims. For the steady states, HR and $\dot{V}{\text{O}}_{2}$ were found in a similar range with a slightly better predictability of $\dot{V}{\text{O}}_{2}$ at the 80 W level. *r*_PRBS_ in Table 1, Res_ME_, and Res_SE_ (Table 2) indicate the best predictability for HR for the PRBS. The tendency to overestimate lower and to underestimate higher CRP values during the PRBS (Fig. 4) can be assigned to restriction of the analysis to a time frame of 150 s as result from the PRBS applied. The alternative would be an extension of the protocol (Khoo 2018, p. 250). However, the intention was to create a feasible, short test, which can be applied to subjects and patients with restrictions regarding exercise duration and intensity.

The main challenge of this analysis was the prediction of the parameter’s behaviour for higher WRs (here 80–175 W). The high correlations (*r*_80–175 W_) are not surprising due to the increasing CRP values. More interesting are the relative residuals. The significantly lowest mean (Res_ME_) was found for $\dot{V}{\text{O}}_{2}$, slightly higher variations were observed for HR and the largest variations were demonstrated for $\dot{V}_{{\text{E}}}$ and $\dot{V}{\text{CO}}_{2}$.

With increasing WR the predictability becomes less accurate, especially for $\dot{V}_{{\text{E}}}$ and $\dot{V}{\text{CO}}_{2}$. This might be related to the increase of anaerobic metabolism and additional ventilatory drives compared to the moderate WR during PRBS. Further research might be focussed on the relation with ventilatory thresholds (see Galán-Rioja et al. 2020 for an overview).

### Dynamic linearity

The CRP impulse responses *h*_TD_(*t*) deviate from responses of first-order systems and its time constants as reported in the literature (e.g. Linnarsson 1974; Scheuermann et al. 2002). The shape of the HR *h*_TD_(*t*) seems to be close to a first-order system with a 40 s time constant, even though this would be a much slower response compared with other published data with *τ* < 30 s (e. g. Linnarsson 1974; Tiedt et al. 1975; DeLorey et al. 2004). For the respiratory parameters, as $\dot{V}{\text{O}}_{2}$, $\dot{V}{\text{CO}}_{2}$, and $\dot{V}_{{\text{E}}}$ show a constant decrease after their maxima which is untypical for first order systems per se. Therefore, these are important arguments to characterize the kinetics of CRP without explicit models.

Although the relative residuals for Pred_TD_ (Table 2) for the $\dot{V}{\text{O}}_{2}$ data indicate impressively low deviations from measured data, this parameter like all others showed significant trends towards higher values (Fig. 4). This is an indication for the need of a non-linear model which will be the subject for further development. The reasons for these non-linearities should be different for each CRP. Commonly, PRBS testing comprises both on- and off-stimuli. The linear modelling implies symmetrical on- and off-responses which might not meet physiological reality. For HR, regulation of blood pressure and vasomotor control are potential physiological explanations for asymmetries. $\dot{V}{\text{O}}_{2}$ and $\dot{V}{\text{CO}}_{2}$, as pulmonary parameters, might be influenced by non-linearities from venous transport. The portion of anaerobic metabolism leads to additional ventilatory drives with a strong influence on both, $\dot{V}_{{\text{E}}}$ and $\dot{V}{\text{CO}}_{2}$. It can be speculated that such non-linearities are caused by the influence from the circulatory system (Eßfeld et al. 1991). Therefore, at least the non-linearities of $\dot{V}_{{\text{E}}}$ and $\dot{V}{\text{CO}}_{2}$ might be influenced by aerobic capacity and/or aerobic-anaerobic threshold. This might also be true for higher WRs for HR and $\dot{V}{\text{O}}_{2}$.

## Limitations

The PRBS protocol applied in this study should be adapted to the abilities and capacities of the subjects. As demonstrated by Kusenbach et al. (1999) maximal WR in the PRBS protocol may be adapted to specific requirements of the tested subjects. Other modifications were already shown by applications with other modes of exercise, e.g. treadmill (Koschate et al. 2016a, b) or arm cranking exercise (Drescher et al. 2015). With a certain loss of reliability, the PRBS could be shortened, e.g. from 600 to 450 s, which might be a compromise for specific groups.

For $\dot{V}{\text{O}}_{2}$ and $\dot{V}{\text{CO}}_{2}$ analysis, some further improvements might be expected if algorithms are applied to model the alveolar gas exchange more precisely (Koschate et al. 2019a, b).

## Conclusions

In summary, the PRBS test, and the resulting kinetics, described by the impulse response, are qualified to describe the kinetics and to assess steady states within the applied WR range. At least below the ventilatory threshold, $\dot{V}{\text{O}}_{2}$ and HR responses to incrementally increasing exercise intensities can be sufficiently predicted from the kinetics derived in the PRBS WR range of 30 W and 80 W. This offers the possibility to assess static as well as kinetics information of subjects with a 10 min moderate exercise test. These data can give important information about the actual state of fitness of the subject which can be used to prescribe physical training or to define its success.

## Data Availability

Data available upon request due to privacy/ethical restrictions.

## Abbreviations

${\stackrel{\mathrm{\sim }}{\text{h}}}_{\text{FD}}\left({\text{t}}\right)$: Non-normalized Impulse response function resulting from frequency domain analysis
${\stackrel{\mathrm{\sim }}{\text{h}}}_{\text{TD}}\left({\text{t}}\right)$: Non-normalized Impulse response function resulting from time-domain analysis
${\widehat{\text{WR}}}_{\text{PRBS}}\left({\text{t}}\right)$: Approximation of WR in the PRBS interval
*ϕ*_*xx*_(*j**ω*): Power-spectral density
*ϕ*_*xx*_(*t*): Auto-correlation function
*ϕ*_*xy*_(*j**ω*): Cross-spectral density
*ϕ*_*xy*_(*t*): Cross-correlation function
*δ*: Constant time for a PRBS level
*a*: Gain
ACF: Auto-correlation functions
*b*: Offset
BMI: Body mass index
CCF: Cross-correlation function
CRP: Cardiorespiratory parameters
CRP_PRBS_(*t*): CRP during the PRBS interval
FD: Frequency domain
*h*(*t*): Normalized impulse response function
HERA: Human Exploration Research Analogue
*H*_FD_(*j**ω*): Transfer function
*h*_FD_(*t*): Normalized impulse response function resulting from frequency domain analysis
HR: Heart rate
*h*_TD_(*t*): Normalized impulse response function resulting from time-domain analysis
PRBS: Pseudorandom binary sequence
